# Personalized Medicine for Orthopaedic Disorders

**DOI:** 10.3390/jpm13111553

**Published:** 2023-10-30

**Authors:** Nan Jiang

**Affiliations:** Division of Orthopaedics & Traumatology, Department of Orthopaedics, Nanfang Hospital, Southern Medical University, Guangzhou 510515, China; hnxyjn@smu.edu.cn

Orthopaedic disorders, also known as musculoskeletal disorders (MSDs), refer to diseases or injuries of the bone, joint, cartilage, muscle, tendon, nerve, and spinal disc. As MSDs display characteristics of complexity and high heterogeneity, clinical diagnosis is sometimes difficult and treatment is often tricky. Take osteomyelitis as an example, which presently still poses great challenges to orthopaedic surgeons while clinical efficacy remains unsatisfactory. Meanwhile, such a disorder aggravates the economic burden of the patients and their families. According to a recent survey, the median healthcare cost of patients with post-traumatic osteomyelitis was almost five-fold higher than that for those without infection [1]. In addition, patients with osteomyelitis experienced high risks of comorbidity, such as epilepsy [2], diabetes mellitus [3], and even depression [4]. These suggest great influences of osteomyelitis on the patients, both physically and psychologically.

In order to keep up with the latest knowledge in the fields of MSDs, this Special Issue was set up with the aim of collecting current investigations focusing on MSDs. In total, we received twenty-three submissions and after evaluations by the editorial office staff, myself, and the peer reviewers, and we finally accepted eleven papers, including six articles, two communications, two study protocols and one perspective. Here, I briefly introduce the eleven articles in this Special Issue.

In a prospective study, Lindemann et al. [5] investigated the therapeutic effectiveness of CT-assisted infiltration with respect to pain, function, and life quality between two different kinds of injections (periradicular infiltration, PRI versus facet joint capsule infiltration, FJI) in chronic complaints. The outcomes of 87 patients from FJI group and 109 patients from PRI group demonstrated that PRI, not FJI, is an easy and suitable strategy to provide a durable therapeutic value for patients with chronic radicular pain and related low back pain.

In a retrospective study, Hu et al. [6] compared the efficacy between minimally invasive transforaminal lumbar interbody fusion (MIS-TLIF) and traditional open transforaminal lumbar interbody fusion (OPEN-TLIF) for the management of two-level lumbar degenerative disorders. Outcomes revealed that similar results were obtained regarding the life quality after surgery, radiological findings, risks of muscle injury and other types of complications between the two groups. However, patients that received MIS-TLIF had a longer surgical time and more radiation exposures during surgery than those by OPEN-TLIF. In addition, patients in the MIS-TLIF group also experienced a higher risk regarding the pedicle screws deviating laterally out of the vertebral body. Thus, they recommended the OPEN-TLIF technique for the treatment of two-level lumbar degenerative diseases.

In a prospective, blinded study, Schwesig et al. [7] compared the accuracy of the internal rotation and Shift (IRO/Shift) test with the Jobe test for evaluation of the recovery following arthroscopic repair of the superior rotator cuff at 3 and 6 months after surgery. Based on the outcomes of the 51 patients included, the authors concluded that the accuracy of the IRO/Shift test was better than the Jobe test, though the accuracy of both tests improved between 3 and 6 months after surgery.

In a prospective study, Chen et al. [8] introduced a novel method, named devascularized bone surface culture (BSC), with comparison to the traditional tissue sampling culture (TSC), for identifying osteomyelitis-related microorganisms. According to the results of 51 patients, the authors reported that the detectable rate following BSC was relatively higher than that of the TSC (75% vs. 59%, *p* = 0.093). Meanwhile, the median culture time following BSC was significantly shorter than the TSC (1 day vs. 3 days, *p* < 0.001). Therefore, they concluded that BSC may be better than TSC for detecting osteomyelitis-related microorganisms.

In a prospective, non-randomized, interventional trial, Molnar et al. [9] assessed clinical efficacy following the use of autologous conditioned adipose tissue (ACA) and leukocyte-poor PRP (LP-PRP) in patients with mild to moderate knee osteoarthritis (KOA). The outcomes of 16 patients revealed that the combination of ACA and LP-PRP, as a minimally invasive approach, offered excellent improvements in symptoms among the patients with mild to moderate KOA.

In a cadaveric and computational-analysis-based study, Wysocki et al. [10] examined the differences of femoral structures and geometric properties between healthy and osteoporotic bones. The outcomes of 42 cadaveric CT data showed that statistical differences were found regarding multiple geometric properties between healthy and osteoporotic femoral bones. Such differences of the geometric properties are evident locally. In addition, this study also demonstrated the feasibility of using CT-scans-based 3D models for analysing the differences in femoral shapes and related biomechanical properties.

In a bioinformatics analysis- and machine-learning-based study, Xu et al. [11] conducted a bioinformatic analysis of immune cell infiltration in cartilage and synovium of OA, and identified three potential risk genes, *PTGS1*, *HLA-DMB* and *GPR137B*. These genes were found to interact with the immune system in OA, which provides a feasible direction for future drug research and development for this disorder.

In a prospective interventional study, Yu et al. [12] assessed the efficacy and safety of using autologous micro-fragmented adipose tissue (MF-AT) for improving joint function and repairing cartilage in KOA patients. Based on the outcomes of 20 patients, the authors concluded that autologous MF-AT can help improve the knee function and relieve pain without adverse events. Nonetheless, the improved knee function failed to be sustained, with the best results occurring at 9 to 12 months after intervention and the cartilage regeneration still needing to be further explored.

In a retrospective multicentre study conducted in six healthcare centres in Eastern China, Zhong et al. [13] characterized the bacterial spectrum following open fractures and analysed the situations of bacterial resistance to antibiotics from 2015 to 2017. The data of 1348 patients showed that the positive rate of culture following open fractures was about 55%, 59% of which were detected in grade III fractures. It was noted that approximate 73% of the identified pathogens were sensitive to prophylactic antibiotics, with quinolones and cotrimoxazole showing the lowest resistant rates. Based on the findings, the authors suggested supplemental antibiotics to cover for Gram-negative bacteria for grade II open fractures.

In a perspective study, Maggioni et al. [14] systematically introduced patellar instability, including the classification of patellofemoral disorders, principal factors of instability, patellofemoral instability, diagnosis, treatment options and follow-up for primary dislocation. This can help readers better understand the clinical diagnosis and treatment of patellar instability.

In a protocol of a randomized controlled trial (RCT), Molina-García et al. [15] focused on the clinical efficacy of foot orthoses (FO) for the treatment of paediatric flat foot (PFF). Through this RCT, they aimed to determine if personalized FO, combined with a specific exercise regimen, could produce results similar to or even better than only specific exercises. The authors hypothesized that such a combination can better improve the signs and symptoms of PFF.

In summary, the studies included in this Special Issue cover different areas regarding epidemiology, diagnosis, and treatment of different types of MSDs. In the future, more investigations with high-level evidence are required to drive our progress toward improving our knowledge to meet the challenges and future prospects of MSDs.
